# Maternal Birth Weight From *Maternal and Child Health Handbooks* Predicts LGA Neonates Better Than Maternal Parameters in Pregnancy

**DOI:** 10.1155/jp/4500495

**Published:** 2025-02-16

**Authors:** Kaname Dateoka, Suguru Mabuchi, Yuiko Nagamine, Takanari Arai, Masayoshi Hashimoto

**Affiliations:** ^1^Department of General Medicine, Keiju General Hospital, Nanao, Ishikawa, Japan; ^2^Department of General Medicine, Institute of Science Tokyo, Bunkyo, Tokyo, Japan; ^3^Department of Public Health, Institute of Science Tokyo, Bunkyo, Tokyo, Japan; ^4^Department of Obstetrics and Gynecology, Keiju General Hospital, Nanao, Ishikawa, Japan

## Abstract

**Objective:** This study is aimed at evaluating maternal birth weight, recorded in Japan's *Maternal and Child Health Handbooks*, as a predictor for large for gestational age (LGA) neonates compared to traditional pregnancy factors.

**Methods:** In this retrospective study, we analyzed maternal and neonatal data from 374 singleton, full-term pregnancies at Keiju General Hospital (2017–2020). Maternal birth weight was obtained from Japan's Maternal Child Health Handbooks, and fasting plasma glucose was measured during the 75-g oral glucose tolerance test (OGTT). Logistic regression models assessed the predictive contributions of maternal birth weight and fasting plasma glucose, adjusted for maternal and pregnancy factors.

**Results:** Among 374 patients, 9.8% of neonates were classified as LGA. This group had a higher proportion of a family history of diabetes (*p* = 0.04) and greater maternal height (*p* = 0.01), pre-pregnancy weight (*p* = 0.004), weight before delivery (*p* = 0.03), and maternal birth weight (*p* = 0.001) than the non-LGA group. Multivariate analysis showed that maternal birth weight remained a significant predictor of neonatal birth weight after adjusting for other risk factors (odds ratios: 2.92 for maternal birth weight between 3500 and 3999 g and 4.77 for birth weight ≥ 4000 g).

**Conclusion:** This study suggests the potential of incorporating maternal birth weight to improve LGA risk prediction. These findings provide foundational data for further research into the integration of maternal birth weight in risk assessment models and its potential clinical applications.

## 1. Introduction

Birth weight above the 90th percentile for sex and gestational age is defined as large for gestational age (LGA), with a prevalence of approximately 10% globally [1–3]. LGA is associated with short-term complications like delivery trauma, cesarean section, and neonatal respiratory issues [4–6], as well as long-term health risks, including obesity, hypertension, cardiovascular diseases, Type 2 diabetes, and psychiatric disorders [7–10]. Identifying maternal risk factors is crucial for predicting birth weight, informing delivery planning, and enhancing informed consent processes.

Known risk factors include gestational diabetes, pre-pregnancy obesity, excessive gestational weight gain, and a history of macrosomia [6, 11, 12], with prediction attempts incorporating maternal blood glucose, lipids, and fetal ultrasound [13–16]. Several studies have reported that fasting plasma glucose (FPG) during the 75-g oral glucose tolerance test (OGTT) is strongly associated with neonatal birth weight, with FPG showing the highest correlation [17, 18]. Similarly, maternal birth weight has been reported to correlate with neonatal birth weight [19, 20]; however, comparisons with other established risk factors remain limited.

This study evaluates the effectiveness of maternal birth weight, recorded in Japan's *Maternal and Child Health (MCH) Handbooks*—comprehensive health records covering prenatal to childhood stages [21]—by comparing it with existing risk factors. Maternal birth weight was collected by requesting mothers to bring their own *MCH Handbooks* during prenatal checkups and analyzed alongside pregnancy parameters using logistic regression to calculate adjusted odds ratios (ORs). This study highlights the importance of incorporating maternal birth weight into traditional risk factors, aiming to enhance LGA prediction and contribute to better clinical decisions regarding delivery timing and methods.

## 2. Method

### 2.1. Study Design

In this retrospective study, we analyzed existing medical records and compared maternal parameters of LGA and non-LGA neonates. The patients in this study were singleton and full-term pregnant women who underwent a 75-g OGTT and delivered at Keiju General Hospital in Nanao, Ishikawa, Japan, from April 2017 to July 2020. Those with multiple births, fetal anomalies, preterm delivery, delivery at another hospital, missing data for the 75-g OGTT, or missing other collection data were excluded. Data were collected from electronic medical records and maternal maternity records.

### 2.2. Sample Size

Based on previous studies, the prevalence of LGA was assumed to be 10% [1–3], and the sample size required for logistic regression analysis to compare the contributions of maternal birth weight and FPG was calculated. The calculation was performed with a significance level of 5% and a power of 80% [22], resulting in an estimated required sample size of approximately 340.

### 2.3. Data Collection

We recorded the following from the electronic medical records: basic information on the mother (maternal height, self-reported pre-pregnancy weight, number of days of labor, number of weeks of labor, mode of labor, spontaneous onset of labor, previous LGA delivery, method of pregnancy, family history of diabetes, obstetric and nonobstetric maternal complications, and number of weeks when the 75-g OGTT was performed), information on the neonate (sex and birth weight), and laboratory data (75-g OGTT blood glucose insulin concentrations at fasting and at 30, 60, and 120 min). We collected maternal birth weight data from the mothers' *MCH Handbooks*. Gestational weeks were determined mainly by an ultrasound examination in the first trimester of pregnancy. LGA was defined as greater than or equal to the 90th percentile, using the standard birth weight values by gestational age and sex developed by the Japan Pediatric Society.

### 2.4. Statistics

A logistic regression analysis was performed using Stata BE (Stata Corp LLC, College Station, Texas, United States) with the presence or absence of LGA (LGA and non-LGA groups) as the objective variable. For continuous variables among the independent variables, normality was assessed by visual inspection using histograms and the Kolmogorov–Smirnov test for normality. Logistic regression analysis was performed to compare the independence and relative contributions of maternal birth weight and FPG to the risk of LGA. A stepwise method was used to construct models incrementally through statistical evaluation, allowing for the assessment of changes in the contributions of explanatory variables at each stage. As a result, the following three models were tested:

Model 1: Basic risk factors were analyzed, with maternal age and parity included as covariates.

Model 2: In addition to the covariates in Model 1, family history of diabetes, history of delivering an LGA baby, maternal height, pre-pregnancy weight, weight just before delivery, and FPG were included as covariates.

Model 3: In addition to the covariates in Model 2, fasting insulin levels, as well as 75-g OGTT glucose and insulin levels at 30, 60, and 120 min, were included as covariates.

Before performing logistic regression analysis, Pearson's correlation coefficients were calculated to evaluate the relationships between independent variables. Multicollinearity was assessed, and it was confirmed that there was no multicollinearity affecting the final models (Models 1–3). A *p* value <0.05 was considered statistically significant.

This study was conducted with the approval of the ethics committee of Keiju General Hospital (Approval No. 2022-4-1, October 24, 2022) and the Institute of Science Tokyo (Approval No. M2022-100, November 12, 2022) and complied with the protection of personal information and anonymization. The consent of the patients was based on the characteristics of a retrospective study. Although individual consent forms were not required for the analysis of existing medical records, opt-outs were obtained at our hospital to ensure that information regarding the purpose and methods of the study was appropriately communicated to medical institutions and other relevant parties. Results and data from the study were anonymized so that individual patients could not be identified, and confidential information was protected.

## 3. Results

**Figure 1:**
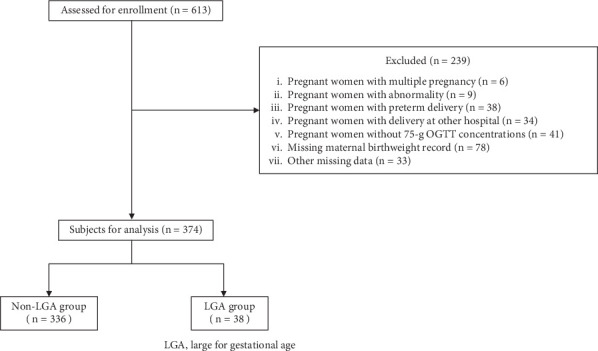
Study enrollment flowchart. Abbreviation: LGA, large for gestational age.

During the study period, 613 patients were identified, of which 535 (87%) submitted their *MCH Handbooks* with their birth records. Based on the criteria (Figure 1), a total of 374 patients were included in the final analysis. Tables 1 and 2 show the basic information of mothers and neonates. The number of LGA neonates was 38 (9.8%). There were no significant differences in maternal age, the number of deliveries, or the mode of delivery between the LGA and non-LGA groups. However, the LGA group had a higher proportion of a family history of diabetes than the non-LGA group (*p* = 0.04). Additionally, maternal height (*p* = 0.01), pre-pregnancy weight (*p* = 0.004), weight before delivery (*p* = 0.03), and maternal birth weight (*p* = 0.001) were significantly higher in the LGA group than in the non-LGA group.  Table 3 presents the results of the logistic regression analysis conducted for Models 1–3. In Model 1, the OR was 3.49 (95% confidence interval (CI), 1.60–7.60) when the mother's birth weight was between 3500 and 3999 g, and the OR was 5.55 (95% CI, 1.54–19.91) when the mother's birth weight was ≥ 4000 g. In Model 2, the OR was 2.92 (95% CI, 1.27–6.67) when maternal birth weight was between 3500 and 3999 g, and the OR was 4.77 (95% CI, 1.22–18.58) when maternal birth weight was ≥ 4000 g. These ORs were higher than those for the other risk factors. In Model 3, the OR was 2.33 (95% CI, 0.96–5.62) when the mother's birth weight was between 3500 and 3999 g, and the OR was 4.56 (95% CI, 1.05–19.63) when the mother's birth weight was ≥ 4000 g. These ORs for maternal birth weight were higher than those for the other risk factors.

## 4. Discussion

While many studies have investigated LGA prediction using maternal parameters during pregnancy, maternal birth weight is not listed among the established risk factors. This study highlights the significance of maternal birth weight as an independent predictor for LGA, particularly when compared to traditional parameters such as maternal glucose levels during pregnancy. The main findings of this study were as follows. First, maternal birth weight remained significantly associated with neonatal birth weight even after adjusting for other risk factors, including plasma glucose concentrations. Second, FPG during the 75-g OGTT was the only glucose-related parameter significantly associated with LGA outcomes. Third, the high data collection rate achieved using Japan's *MCH Handbooks*—a unique, comprehensive health record system—enabled reliable analysis.

In this study, maternal birth weight remained significantly associated with neonate birth weight even after adjusting for other risk factors such as plasma glucose concentrations. Our finding that maternal birth weight was associated with neonatal birth weight is similar to that of previous studies [19, 20]. In this study, maternal birth weight was more strongly related to the birth weight of the mother's offspring than to other risk factors, including maternal plasma glucose concentrations. The relationship between maternal birth weight and neonatal birth weight was first described in 1983, and more recently, the association between maternal birth weight and the long-term prognosis of infants has been reported [23, 24]. The mechanism of these associations is not fully understood, although it may be attributed to genetic attributes, environmental exposures, and intergenerational socioeconomic factors. Although the maternal age at birth has increased over time in developed countries, maternal birth weight affects neonatal birth weight [19, 20], as observed in the present study, and is considered an independent influencing factor, regardless of maternal age at birth.

Next, FPG was significant in predicting LGA neonates, while the association with other time-point glucose values was limited. FPG is thought to reflect the mother's basal metabolic state, and this result aligns with previous studies [17, 25]. Insulin is known to play a crucial role during pregnancy, promoting maternal hypertriglyceridemia, altering lipid transport to the fetus [26], and upregulating other placental transport pathways as the most critical growth hormone [27]. Resistance to basal insulin primarily drives the elevation in FPG. The 75-g OGTT is a glucose-only challenge that does not represent the typical dietary intake of pregnant women. Consequently, plasma glucose values at 30 min, 1 h, and 2 h primarily reflect transient fluctuations rather than the mother's habitual nutritional status. Compared to FPG levels, postload glucose increases are less suitable for predicting LGA.

These findings suggest that 75-g OGTT values, other than FPG, may have limited utility in predicting LGA. While the OGTT is invaluable for diagnosing gestational diabetes mellitus, this study implies that incorporating maternal birth weight, rather than relying on postload OGTT values, could provide a more effective approach to predicting LGA outcomes.

**Figure 2:**
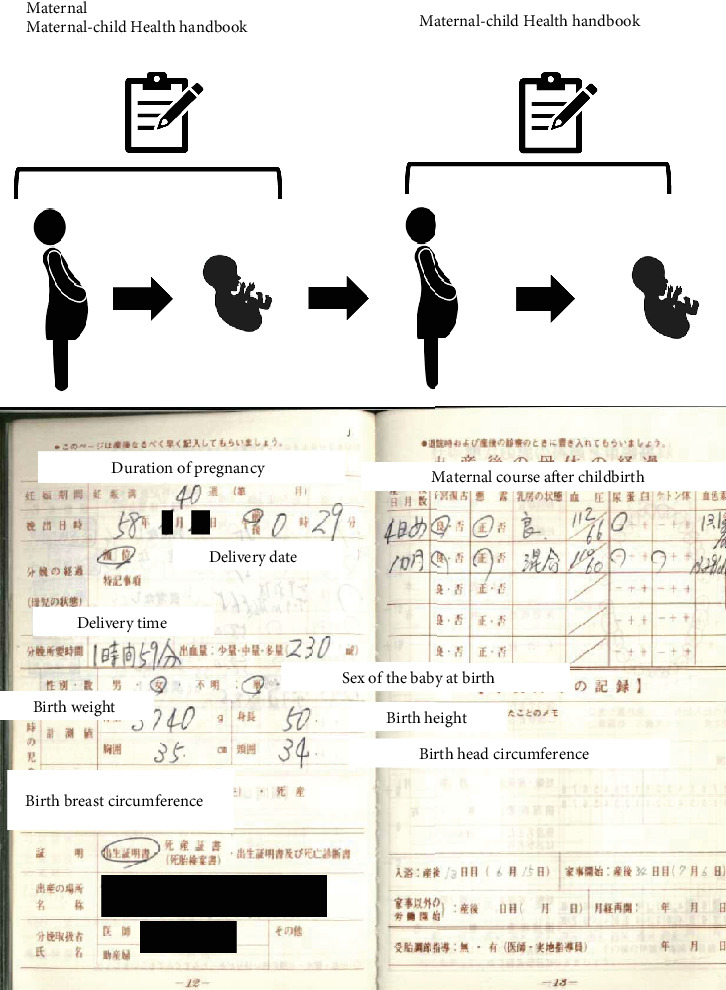
*Maternal and Child Health (MCH) Handbook*. This handbook is distributed in Japan when a pregnancy notification is submitted to the local government office. The handbook shown in this photo is the one the mother's own mother—her grandmother—received when she was pregnant. The handbook includes medical information, such as pregnancy progress, events during childbirth, birth details, postpartum recovery, vaccinations during infancy and early childhood, and growth charts. Additionally, it provides a section for the mother to write messages to her baby during pregnancy.

Finally, the use of Japan's *MCH Handbooks* allowed for an outstandingly high rate of data collection, demonstrating their effectiveness as a detailed and reliable source of health information. Only a limited number of countries collect and manage birth weight data through national databases. In Japan, however, this role is fulfilled by the *MCH Handbooks*. In this study, maternal birth weight information was obtained from the *MCH Handbooks* brought by the participants. The handbook contains comprehensive records of the mother's pregnancy and delivery, as well as the child's growth, development, vaccination history, and other health-related details. In a 2010 report, it was noted that the handbook was being used in over 20 countries worldwide [21], with studies also reporting its effectiveness in helping mothers acquire knowledge during pregnancy and child health care [28]. Of the 613 participants included in this study for data collection, more than 80% provided their *MCH Handbooks* upon our request, allowing us to verify the mothers' birth weights from these records (Figure 2). This high availability was likely due to the cultural norm of presenting the handbook during pediatric visits and its sentimental value, as it often includes spaces for parents to document their thoughts during pregnancy. While the handbook's paper-based format may present a limitation, digital records, which are often linked to individuals, are currently less suitable for intergenerational information sharing. Nonetheless, the *MCH Handbooks*, with its detailed records, hold significant potential as a valuable resource for predicting future health risks across generations.

A limitation of this study is the absence of paternal birth weight data, which has been reported to influence neonatal birth weight, albeit less strongly than maternal birth weight [20, 29]. Additionally, body composition data for neonates, particularly for those born to mothers with gestational diabetes mellitus, were not included, despite reports that such neonates may exhibit higher body fat mass [30, 31]. Furthermore, the sample size of 340 participants may not have provided sufficient statistical power to detect smaller effects. Adjustment variables were selected based on existing literature, but it is possible that other relevant factors were excluded. Lastly, the dataset was geographically limited, which could restrict the generalizability of the findings. Future studies should address these limitations by including a more diverse sample, additional variables, and paternal and neonatal data to strengthen predictive models.

## 5. Conclusion

The findings of this study underscore the importance of considering maternal birth weight as a routine factor in LGA prediction models, particularly in contexts where *MCH Handbooks* or similar health records are available. The incorporation of maternal birth weight alongside existing parameters could significantly enhance the accuracy of neonatal weight predictions, thereby improving clinical decision-making processes, including the timing and mode of delivery. Future research should explore the applicability of similar record systems in other countries and evaluate their potential to support intergenerational health data collection and risk prediction.

## Figures and Tables

**Table 1:** Clinical characteristics of the women in each neonatal birth weight group.

**Variables**	**Non-LGA**	**LGA**	**p** ** value**
	**(** **n** = 336**)**	**(** **n** = 38**)**	
Maternal age (years)	31 (20–44)	32 (20–39)	0.62
Parity			
0	148 (44.0)	19 (50.0)	0.87
1	122 (36.3)	13 (34.2)	
2	50 (14.9)	4 (10.5)	
3	13 (3.9)	2 (5.3)	
4	3 (0.9)	0 (0.0)	
Mode of delivery			
Cephalic vaginal delivery	209 (62.2)	21 (55.3)	0.53
Vacuum delivery	29 (8.6)	6 (15.8)	
Planned cesarean section	57 (17.0)	6 (15.8)	
Emergency cesarean section	41 (12.2)	5 (13.2)	
Neonate's sex			
Female	149 (44.3)	13 (34.2)	0.23
Male	187 (55.7)	25 (65.8)	
Induction of labor			
No	198 (58.9)	23 (60.5)	0.85
Yes	138 (41.1)	15 (39.5)	
Family history of diabetes			
No	153 (45.5)	24 (63.2)	0.04
Yes	183 (54.5)	14 (36.8)	
History of giving birth to an LGA infant			
No	314 (93.5)	35 (92.1)	0.75
Yes	22 (6.5)	3 (7.9)	
Standing work for ≥ 3 h			
No	156 (46.4)	15 (39.5)	0.41
Yes	180 (53.6)	23 (60.5)	
Mode of pregnancy			
Natural pregnancy	275 (81.8)	33 (86.8)	0.12
Ovulation induction	29 (8.6)	0 (0.0)	
AIH	8 (2.4)	0 (0.0)	
ART	24 (7.1)	5 (13.2)	
Hospital bed rest for ≥ 2 weeks			
No	263 (78.3)	33 (86.8)	0.22
Yes	73 (21.7)	5 (13.2)	
Presence of outpatient dietary guidance			
No	269 (80.1)	26 (68.4)	0.10
Yes	67 (19.9)	12 (31.6)	
Presence of insulin treatment			
No	312 (92.9)	34 (89.5)	0.45
Yes	24 (7.1)	4 (10.5)	
Maternal height (cm)	158.4 (146.5–176.2)	161.2 (151–172)	0.01
Maternal pre-pregnancy weight (kg)	53.8 (38.0–90.8)	58 (43.1–87.4)	0.03
Maternal pre-pregnancy weight (kg)	64.5 (46.0–113.2)	71.2 (53.0–93.7)	0.004
Maternal birth weight (g)	3064 (1454–4620)	3244 (2550–1454)	0.001

*Note:* Data are the mean (standard deviation), *n* (percent), or median (range).

Abbreviations: AIH, artificial insemination by husband; ART, assisted reproductive technology; LGA, large for gestational age.

**Table 2:** Laboratory characteristics of the women in each neonatal birth weight group.

**Variables**	**Non-LGA**	**LGA**	**p** ** value**
	**(** **n** = 336**)**	**(** **n** = 38**)**	
FPG (mg/dL)	80 (66–102)	82 (69–93)	0.02
75-g OGTT 30-min glucose concentrations (mg/dL)	133 (76–209)	127.5 (94–162)	0.32
75-g OGTT 60-min glucose concentrations (mg/dL)	138 (67–227)	136 (93–172)	0.57
75-g OGTT 120-min glucose concentrations (mg/dL)	117 (55–198)	109 (69–150)	0.16
Fasting insulin concentrations (*μ*U/mL)	5.7 (1.4–38.0)	5.9 (2.40–26.3)	0.88
75-g OGTT 30-min insulin concentrations (*μ*U/mL)	49.9 (10.1–200.6)	43.9 (14.1–163.2)	0.32
75-g OGTT 60-min insulin concentrations (*μ*U/mL)	52.7 (6.8–187.9)	49.2 (29.5–232.6)	0.90
75-g OGTT 120-min insulin concentrations (*μ*U/mL)	46.9 (8.7–238.3)	41.1 (6.2–157.3)	0.10

*Note:* Data are the mean (standard deviation), *n* (percent), or median (range).

Abbreviations: FPG, fasting plasma glucose; LGA, large for gestational age; OGTT, oral glucose tolerance test.

**Table 3:** Result of logistic regression analysis across Models 1–3.

**Variables**	**Model 1**			**Model 2**			**Model 3**		
	**OR**	**95% CI**	**p** ** value**	**OR**	**95% CI**	**p** ** value**	**OR**	**95% CI**	**p** ** value**
Maternal birth weight									
3500–3999 g	3.49	1.60–7.60	0.002	2.92	1.27–6.67	0.009	2.33	0.96–5.62	0.03
≥ 4000 g	5.55	1.54–19.91	0.007	4.77	1.22–18.58	0.02	4.56	1.05–19.63	0.04
Maternal age	1.02	0.94–1.09	0.89	1.01	0.93–1.09	0.97	1.01	0.93–1.09	0.95
Parity	0.80	0.51–1.21	0.33	0.76	0.48–1.19	0.24	0.72	0.44–1.16	0.16
Family history of diabetes mellitus				0.49	0.23–1.02	0.05	0.51	0.23–1.09	0.06
History of delivering an LGA infant	—	—	—	0.91	0.22–3.63	0.96	0.91	0.2–3.95	0.98
Maternal height	—	—	—	1.05	0.97–1.12	0.17	1.03	0.95–1.11	0.30
Maternal pre-pregnancy weight	—	—	—	0.93	0.85–1.02	0.3	0.92	0.83–1.01	0.29
Maternal pre-delivery weight	—	—	—	1.08	0.98–1.19	0.23	1.12	1.01–1.25	0.14
Maternal FPG	—	—	—	1.06	0.98–1.13	0.04	1.10	0.99–1.19	0.01
75-g OGTT glucose concentrations at 30 min	—	—	—	—	—	—	0.99	0.96–1.02	0.58
75-g OGTT glucose concentrations at 60 min	—	—	—	—	—	—	1.00	0.96–1.02	0.94
75-g OGTT glucose concentrations at 120 min	—	—	—	—	—	—	0.98	0.95–1.01	0.68
Fasting insulin	—	—	—	—	—	—	0.90	0.75–1.06	0.24
75-g OGTT insulin concentrations at 30 min	—	—	—	—	—	—	1.00	0.98–1.02	0.91
75-g OGTT insulin concentrations at 60 min	—	—	—	—	—	—	1.00	0.98–1.02	0.63
75-g OGTT insulin concentrations at 120 min	—	—	—	—	—	—	1.00	0.97–1.02	0.63

*Note:* Model 1 was adjusted for maternal age and parity. Model 2: In addition to the covariates in Model 1, family history of diabetes, history of delivering an LGA baby, maternal height, pre-pregnancy weight, weight just before delivery, and fasting plasma glucose were included as covariates. Model 3: In addition to the covariates in Model 2, fasting insulin levels, as well as 75-g OGTT glucose and insulin levels at 30, 60, and 120 min, were included as covariates.

Abbreviations: CI, confidence interval; FPG, fasting plasma glucose; LGA, large for gestational age; OGTT, oral glucose tolerance test; OR, odds ratio.

## Data Availability

The data that support the findings of this study are available from the corresponding author upon reasonable request.
